# Clinical and Socioeconomic Predictors of 60‐Day Rehospitalization After Oncologic Head and Neck Surgery

**DOI:** 10.1002/ohn.70164

**Published:** 2026-02-06

**Authors:** Nana‐Hawwa Abdul‐Rahman, Shaum Sridharan, Matthew E. Spector, Carl H. Snyderman

**Affiliations:** ^1^ University of Pittsburgh School of Medicine Pittsburgh Pennsylvania USA; ^2^ Department of Otolaryngology University of Pittsburgh School of Medicine Pittsburgh Pennsylvania USA

**Keywords:** area deprivation index (ADI), emergency department visit, head and neck cancer, hospital utilization, neighborhood socioeconomic disadvantage, readmission

## Abstract

**Objective:**

Evaluate the association between clinical characteristics and neighborhood socioeconomic disadvantage and 60‐day re‐presentation after oncologic head and neck surgery.

**Study Design:**

This retrospective cohort study involved 1,088 patients who underwent oncologic head and neck surgery from August 2012 to November 2024.

**Setting:**

Tertiary academic center.

**Methods:**

The main outcome measure was 60‐day Emergency Department (ED) visit or readmission after primary hospital discharge following surgery. Patient demographics, clinical characteristics, and neighborhood socioeconomic disadvantage, as measured by state‐level area deprivation index (ADI).

**Results:**

Predictors of re‐presentation within 60 days of discharge on univariate analysis include tumor stage 4 (OR: 1.52, 95% Cl: 1.01‐2.31, *P* = .044), longer primary length of stay (OR: 1.03, 95% Cl: 1.01‐1.05, *P* < .001), and residency in the 4th quartile state ADI (OR: 1.54, 95% Cl: 1.03‐2.31, *P* = .037). Multivariable logistic regression identified laryngeal cancer (OR: 1.60, 95% Cl: 1.16‐2.21, 0.004) and discharge with home health or other care facilities (2.22, 95% Cl: 1.67‐2.96, *P* < .001) as predictive factors. Patients from low ADI neighborhoods (Q1‐3) were more likely to be discharged home, whereas those from high ADI neighborhoods (Q4) where almost twice as likely to be discharged with home health or to care facilities than home alone (18.70% vs 10.29%, *P* = < .001).

**Conclusion:**

Our study demonstrates that clinical and systemic factors contribute to re‐presentation to the hospital after major oncologic head and neck surgery. Addressing these factors through targeted policies and institutional initiatives may help mitigate the morbidity and cost associated with representation.

Unplanned rehospitalization has detrimental implications for clinicians, hospitals, policymakers, and patients.^1^, ^2^, ^3^ In oncologic head and neck surgery, approximately 1 in 5 patients are re‐hospitalized, costing an average of $15,916 per readmission.^4^ Studies have linked patients comorbidities, cancer site, tumor stage, and postoperative complications with rehospitalization.^4^, ^5^, ^6^ Despite this knowledge, rehospitalization rates remain as high as 19.4%.^4^ It is possible that non‐clinical and social factors, such as neighborhood‐level socioeconomic disadvantages may contribute to rehospitalization after surgery.^7^

Neighborhood socioeconomic disadvantages encompass factors such as low income, suboptimal living conditions, limited educational attainment, and inadequate access to healthcare, among other factors.^1^, ^8^ In head and neck surgery, socioeconomic factors like low household income, Black and Asian race, and Medicaid insurance are linked to high readmission rates.^4^, ^5^ While these factors are important when evaluating a patients' readmission risk, assessing each factor for individual patients in a clinical setting may be time‐consuming to integrate into clinical practice. The *area of deprivation index* (ADI) is an objective measure that provides a composite assessment of neighborhood socioeconomic disadvantage using 17 US Census indicators related to income, housing, education, employment status, and access to resources, among other factors.^9^, ^10^ In other surgical and non‐surgical fields, a high ADI has been shown to correlate with rehospitalization and Emergency Department (ED) visits.^11^, ^12^, ^13^ In the head and neck literature, high ADI is associated with postoperative complications, delayed adjuvant treatment, and high psychologic distress.^14^, ^15^, ^16^ By utilizing a composite objective measure of neighborhood‐level socioeconomic disadvantage, this study aims to determine whether ADI predicts 60‐days hospital re‐presentation after oncologic head and neck surgery.

## Methods

### Study Design and Population

This retrospective cohort study included 1088 patients that underwent oncologic head and neck surgery at our institution from August 2012 to November 2024. Data were obtained from our institutions head and neck cancer registry. This study was approved by the University of Pittsburgh's Institutional Review Board (STUDY20050058).

### Outcome Measures

Our main outcome of interest was readmission/ED‐visit within 60 days after primary discharge following oncologic head and neck surgery. Due to the complex postoperative care and delayed discharge for patients, 60 days re‐presentation was chosen to capture all ED visits and readmissions that could be related to primary admission. Primary length of stay (PLOS) was defined as the number of days the patient spent in the hospital after their surgery.

### Variables

ADI for each patient was calculated using the Neighborhood Atlas from the University of Wisconsin‐Madison School of Medicine and Public Health.^10^ ADI is a composite measure of income, educational attainments, employment status, and housing quality. State ADI was used for analysis since most patients were residents of Western Pennsylvania and therefore had the same national ADI. State ADI's range from 1 (most affluent neighborhoods) to 10 (most deprived neighborhoods). Other variables of interest include patient demographics (age at surgery, sex, and race), tumor stage, tumor site, PLOS, readmission length of stay, discharge destination, and reason for readmission/ED‐visits. Reasons for readmission were grouped into surgical site complications (which includes abscess, cellulitis, fistula, chyle leak, and hematoma) and nonsurgical site complications (which includes feeding tube issues, tracheostomy issues, respiratory failure, and medical conditions such as dehydration and dyspnea).

### Statistical Analysis

Statistical analyses were conducted using STATA, version 18 (StataCorp). An alpha level of <0.05 was considered statistically significant. Descriptive statistics were used to summarize the demographics of the patient population. Chi‐square tests and Fisher exact tests were used to analyze categorical variables. Independent *t*‐tests were used to analyze continuous variables. As in prior studies, State ADI was divided into quartiles based on our cohort distribution and analyses were performed comparing our cohorts lowest ADI quartiles (1st‐3rd quartile ADI: 1‐8) to the top ADI quartile (4th quartile ADI: 9‐10).^17^ Multivariable logistic regression was conducted to identify independent predictors of hospital re‐presentation. Exploratory multivariable logistic regression was performed, and stepwise selection was used to construct the final model which included the variables: tumor site, discharge location, and state ADI. Results of the multivariable logistic regression are presented as odds ratio‐OR (95% confidence interval [95% Cl], *P*‐value). Multicollinearity was assessed, and the mean variance inflation factor for all models was below 5.

## Results

Our cohort of 1088 patients had an average age of 60 ± 11.60 years. Most patients were males (70.68%, n = 769), and White (92.83%, n = 1010). Overall, 60‐day readmission rates were 19.30% (n = 210) and ED‐visits were 14.89% (n = 162) for a total bounce‐back rate of 34% (Table 1). Of the patients that re‐presented to the hospital, 34.68% (n = 129) were related to surgical site complications and 65.32% (n = 243) were related to non‐surgical site complications. (Supplemental Figure S1, available online). The median length of stay after surgery was 7 days (IQR: 3‐11). The median time interval from discharge to readmission was 10 days (range: 1‐56 days) for those with surgical site complication and 20 days (range: 7‐60 days) for those with nonsurgical site complications. Early re‐presentation, that is, within 7 days after discharge, was related to surgical site complications rather than nonsurgical site complications (94.87% (n = 37) vs. 5.13%, n = 2, p < .001) (Supplemental Table S1, available online).

**Table 1:** Descriptive Statistics and Patient Presentation

Variables	Total N = 1088	Readmission and ED presentation, Yes n = 370	Readmission and ED presentation, No n = 715
Mean (SD)			
Age at diagnosis (years)	60 (12)	60 (11)	61 (12)
Median (IQR)			
Primary length of stay	7 (3‐11)	8 (4‐13)	7 (3‐10)
Readmission length of stay	6 (3‐13)	6 (3‐13)	
Time interval from discharge to readmission/ED‐visit	16 (10‐27)	16 (10‐27)	
State ADI	7 (5‐9)	7 (5‐9)	7 (5‐9)
National ADI	73 (57‐86)	73 (56‐88)	73 (57‐85.5)
N (%)			
Sex			
Male	769 (70.68)	254 (68.28)	515 (71.93)
Female	319 (29.32)	118 (31.72)	201 (28.07)
Race			
White	1010 (92.83)	342 (92.43)	668 (93.43)
Black	46 (4.23)	20 (5.40)	26 (3.64)
Asian	6 (0.55)	2 (0.54)	4 (0.56)
T Stage			
1	139 (12.78)	41 (11.08)	98 (13.71)
2	214 (19.67)	62 (16.76)	152 (21.26)
3	211 (19.39)	67 (18.11)	144 (20.14)
4	436 (40.07)	170 (45.95)	266 (37.20)
Unknown	88 (8.09)	30 (8.11)	55 (7.69)
Site			
Hypopharynx	32 (2.94)	13 (3.49)	19 (2.65)
Larynx	201 (18.47)	90 (24.19)	111 (15.50)
Nasopharynx	95 (8.73)	35 (9.41)	60 (8.38)
Oral Cavity	489 (44.94)	159 (42.74)	330 (46.09)
Oropharynx	271 (24.91)	75 (20.16)	196 (27.37)
Discharge location			
Home	826 (75.92)	243 (65.32)	583 (81.54)
Intermediate care	42 (3.86)	22 (5.91)	20 (2.80)
Home Health	16 (1.47)	6 (1.61)	10 (1.40)
Hospice	1 (0.09)	0 (0.00)	1 (0.27)
Rehabilitation Facility	31 (2.85)	22 (5.91)	9 (1.26)
Nursing Home	172 (15.81)	79 (21.24)	93 (13.01)
Readmission	210 (19.30)		
ED presentation	162 (14.89)		
Reason for readmission/ED presentation			
Surgical site	129 (34.68)		
Nonsurgical site related	243 (65.32)		
State ADI, quartile			
1	351 (33.40)	121 (33.40)	230 (33.05)
2	265 (25.21)	82 (25.21)	183 (26.29)
3	301 (28.64)	92 (28.64)	209 (30.03)
4	134 (12.75)	60 (12.75)	74 (10.63)
National ADI, quartile			
1	271 (25.78)	94 (26.48)	177 (25.43)
2	273 (25.98)	91 (25.63)	182 (26.15)
3	253 (24.07)	70 (19.72)	183 (26.29)
4	254 (24.17)	100 (28.17)	154 (22.13)

Abbreviations: IQR, interquartile range; SD, standard deviation.

As seen in Supplemental Figure S2, available online, state ADI ranged from 1 to 10 with a median of 7 (IQR: 5‐9). The ADI was similar to other head and neck cancer populations reported in the literature^15^ but significantly higher than national means for rural and urban populations.^18^ Patients of African American and Asian race were more likely to reside in high ADI neighborhoods (African American and Asian: 70.83% vs White Americans: 39.63%, *P* < .001) (Supplemental Table S2, available online). As shown in Table 2, residents of low ADI neighborhoods (Q1‐3) were more likely to be discharged home. Residents of high ADI neighborhoods (Q4– i.e., 9‐10) where almost twice as likely to be discharged with home health or to care facilities than home alone (18.70% vs 10.29%, *P* = < .001).

**Table 2:** Univariate Analysis

	Readmission/representation to ED		
Variables	No (mean [SD])	Yes (mean [SD])	*P*‐value
Age at diagnosis	60.40 (11.37)	61.18 (12.15)	.30
National ADI	68.99 (21.29)	68.83 (23.32)	.91
State ADI	6.47 (2.55)	6.51 (2.78)	.83
	Odds ratio	95% Cl	*P*‐value
Age at diagnosis	1.01	0.99‐1.02	.297
Sex	1.19	0.91‐1.56	.210
Race			
White	Ref		
Black or Asian	1.43	0.81‐2.52	.213
Site			
Hypopharynx	Ref		
Larynx	1.19	0.56‐2.53	.661
Nasopharynx	0.85	0.38‐1.93	.703
Oral cavity	0.70	0.34‐1.46	.347
Oropharynx	0.56	0.26‐1.19	.131
Tumor stage			
1	Ref		
2	0.974	0.61‐1.56	.916
3	1.11	0.70‐1.77	.655
4	1.52	1.01‐2.31	.044*
Discharge location			
Home	Ref		
Intermediate care	2.64	1.41‐4.92	.002*
Home Health	1.44	0.52‐4.00	.485
Hospice	1		
Rehabilitation Facility	5.86	2.66‐12.92	<.001*
Nursing Home	2.04	1.46‐2.85	<.001*
State ADI, quartile			
1	ref	‐	‐
2	0.85	0.61‐1.20	.356
3	0.84	0.60‐1.16	.289
4	1.54	1.03‐2.31	.037*
Primary length of stay	1.03	1.01‐1.05	<.001*
ADI			
Variables	N (%)	N (%)	*P*‐value
Discharge location	Q1‐3	Q4	<.001*
Home	741 (89.71)	85 (10.29)	
Home Health or other facilities	213 (81.30)	49 (18.70)	
ADI (Q1‐2 vs Q3‐4)			
Variables	Odds ratio	95% Cl	*P*‐value
PLOS	1.02	1.004‐1.04	.015*
RLOS	1.01	0.98‐1.03	.635
TLOS	1.01	1.003‐1.02	.044*

Abbreviations: IRR, incidence rate ratio; SD, standard deviation.

* Indicates significance: *P* < .01.

### Predictors of 60‐Day Hospital Readmission and ED‐Visits

#### Univariate Analysis

As shown in Table 2, readmission and ED‐visits were associated with tumor stage 4 (OR: 1.52, 95% Cl: 1.01‐2.31, *P* = .044), longer primary length of stay (OR: 1.03, 95% Cl: 1.01‐1.05, *P* < .001), residency in the 4th quartile state ADI (OR: 1.54, 95% Cl: 1.03‐2.31, *P* = .037), and discharge to intermediate care (OR: 2.64, 95% Cl: 1.41‐4.92, *P* = .002), rehabilitation facility (OR: 5.86, 95% Cl: 2.66‐12.92, *P* =< .001), and nursing home (OR: 2.04, 95% Cl: 1.46‐2.85, *P* = < .001). Patients residing in ADI Q3‐4 had slightly higher odds of having a longer PLOS (OR: 1.02, 95% Cl: 1.004‐1.04, *P* = .015), and total LOS (OR: 1.01, 95% Cl: 1.003‐1.02, *P* = .044) but not readmission (RLOS) (OR: 1.01, 95% Cl: 0.98‐1.03, *P* = .635).

#### Multivariable Analysis

**Figure 1:**
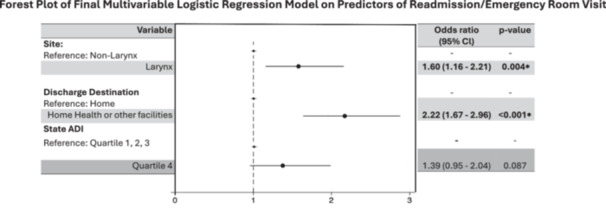
Forest plot of final multivariable logistic regression on predictors of 60‐day representation.

Supplemental Table S3, available online shows the results of the exploratory multivariable logistic regression; Table 3 and Figure 1 show the final model. Independent predictors of increased odds of 60‐days readmission/ED‐visits are patients treated for laryngeal cancer (OR: 1.60, 95% Cl: 1.16‐2.21, 0.004), and those discharged with home health or to other care facilities (2.22, 95% Cl: 1.67‐2.96, *P* < .001).

**Table 3:** Final Exploratory Multivariable Logistic Regression Model: Representation (ED‐Visits and Readmissions)

	Readmission/representation to ED		
	Odds ratio	95% Cl	*P*‐value
Site			
Non‐Larynx, reference	‐	‐	‐
Larynx	1.60	1.16‐2.21	.004*
Discharge location			
Home, reference	‐	‐	‐
Home Health or other facilities	2.22	1.67‐2.96	<.001*
State ADI, quartile			
1, 2, 3, reference	‐	‐	‐
4	1.39	0.95‐2.04	.087

Abbreviation: CI, confidence interval.

## Discussion

### Key Results

This study examined the impact of patient‐level and neighborhood‐level factors on unplanned 60‐days readmission and ED visits following oncologic head and neck surgery. While previous studies have identified patient‐level factors such as wound complications, low household income, and Medicaid insurance, the role of neighborhood‐level factors on rehospitalization has not been explicitly addressed. Our findings reveal that nearly 1 in 3 patients re‐present to the hospital within 60 days of surgery. For those that presented to the ED, 1 in 2 were admitted to the hospital. Patients that re‐presented within 7 days of discharge were more likely to have surgical site complications. We show that both patient‐level and neighborhood‐level factors contribute to unplanned re‐presentation. Specifically, patients with stage 4 tumors, laryngeal cancers, longer PLOS, discharge destination (not home) and residence in neighborhoods with an ADI of 9 and 10 have higher odds of returning to the hospital within 60 days after surgery. Additionally, patients with high ADI were more likely to have longer PLOS and total LOS. Lastly, patients from low ADI neighborhoods were more likely to be discharged home, and vice versa. Our findings suggest that a comprehensive evaluation of patient clinical characteristics and neighborhood‐level socioeconomic disadvantage (as measured by ADI) is important to adequately identify high‐risk surgical patients and reduce unplanned hospital re‐presentation.

### Interpretation and Clinical Implications

#### Tumor Site

Several head and neck cancer studies have shown that certain cancer sites are more likely to re‐present after discharge, particularly laryngeal cancer.^19^, ^20^ Laryngectomy changes airway anatomy and results in respiratory changes, dysphagia, nutritional challenges, and communication difficulties. Postsurgery, management of a permanent tracheostoma and possible feeding tube adds to the burden of care for both patients and their caregivers. Low health literacy on postlaryngectomy care increases the risk of hospital returns especially when complications arise. Studies have demonstrated that postoperative complications such as hematoma, wound infection, pharyngocutaneous fistula, salivary leak, tracheostomy stenosis, and aspiration pneumonia are several reasons patients return to the hospital.^4^ Given these challenges, targeted interventions aimed at enhancing perioperative education during presurgery clinic visits, inpatient stay, and before discharge have proven to be effective at reducing readmission rates.^4^, ^21^, ^22^ A thorough predischarge evaluation of the surgical site can detect rising complications, allowing high‐risk patients to remain admitted for observation, discharged with transitional care, monitored via telemedicine, or scheduled for an earlier post‐surgical clinic visit.^19^

#### Discharge Location

Clinicians should consider discharge destination when determining the appropriate LOS, as discharge destination has been shown to influence readmission risk.^20^ Similar to our study, the literature shows that head and neck cancer patients discharged to nursing homes, with home health, and to other facilities have higher odds of readmission when compared to those discharged home without additional care.^4^, ^20^ An explanation for this finding is that patients' with advanced care needs are more likely to be discharged to facilities or with home health than home alone, thus these patients are at a higher risk of re‐presenting due to the complexity of their condition. Also, healthcare providers at the facilities are more knowledgeable and skilled than the average patient, which may lead to early detection of complications and re‐presentation to the hospital. Legal concerns may also influence providers at these facilities to be cautious and return the patient to the hospital to avoid detrimental legal consequences.

#### Area of Deprivation Index

Notably, on univariant analysis, our study identified an association between ADI and hospital re‐presentation, with patients residing in the most deprived neighborhoods demonstrating increased odds of returning to the hospital. This is a significant addition to the head and neck literature as most attention on risk factors for readmissions and ED visits have focused on factors such as flap failure, necrosis, and wound complications.^4^, ^5^, ^6^ Our study brings attention to neighbourhood‐level factors as a key risk factor for return to the hospital, which aligns with studies in other surgical and non‐surgical fields.^11^, ^23^ Interestingly, while Black and Asian patients were more likely to reside in high ADI neighborhoods, it was ADI but not race that predicted re‐presentation. This finding is consistent with other studies where ADI, but not race, was associated with key health outcomes, such as increased mortality from COVID‐19 and increased mortality in critically ill surgical patients with sepsis.^17^, ^24^ This suggests that factors beyond race, encapsulated within the ADI are predictive of hospital representation. Collectively, these factors can create an environment reflective of structural inequities that drives disparities in readmission and re‐presentation. Deprived neighborhoods often have few healthcare facilities and resources, including primary care providers.^25^ This results in care fragmentation and leaves residents with fewer options besides seeking care at the ED when issues arise.^25^ Interestingly, our study found that patients residing in high ADI neighborhoods had longer PLOS but not RLOS. This suggests that these patients are more likely to present to the ED or be readmitted for minor issues, both of which reflect a shorter readmission LOS.

Financial instability, including low income and unemployment, impacts a patient's ability to effectively manage their health after discharge. Limited financial resources make adhering to a treatment plan challenging.^26^ These may include inability to afford home care services, rehabilitative services, medications, medical supplies, transportation to care facilities, and healthy foods.^27^, ^28^, ^29^ In aggregate, these may hinder recovery and result in care fragmentation, delayed follow‐up, and increase likelihood of complications. Moreover, studies have shown that patients with lower educational attainment demonstrate suboptimal health literacy, which impacts their ability to manage their care effectively.^30^ Head and neck cancer surgery is arguably one of the most complex procedures in otolaryngology, and navigating the recovery process can be challenging for those with limited health literacy. In the postoperative period, patients with low health literacy may have difficulty understanding and implementing written and verbal postsurgical care and discharge instructions, recognizing early signs of complications, adhering to treatment recommendations, or navigating the healthcare system effectively.^31^, ^32^, ^33^, ^34^ Several studies have shown that patients with suboptimal health literacy tend to present to the ED or be readmitted compared to those with sufficient health literacy.^35^ Addressing neighborhood‐level factors such as moving patients from deprived to affluent neighborhoods can improve health outcomes.^36^ Thus, hospitals might consider incorporating measures such as the ADI into risk assessment protocols, to identify patients at risk of adverse health outcomes, such as re‐presentation to the ED or readmission.

ADI could be incorporated into routine preoperative or discharge planning using patients' home addresses to help clinicians identify individuals who may need additional support after surgery. Patients living in highly deprived neighborhoods may benefit from more intentional discharge planning, earlier postoperative follow‐up, closer care coordination, and timely involvement of social work or community‐based resources. ADI‐informed risk assessment could also guide the allocation of limited supportive services (such as home health visits, telehealth check‐ins, transportation assistance, or support with medication access) to patients who are most likely to benefit. Future prospective studies are needed to determine whether ADI‐guided interventions can reduce hospital re‐presentation and improve equity in postoperative outcomes following head and neck cancer surgery. Additionally, addressing health literacy could play a key role in reducing hospital readmission and ED visits. Incorporating strategies highlighted by Pugh et al. prior to discharge, including calculating readmission risk or assessing a list of risk factors, increasing patient education about their diagnoses, self‐management, and medications throughout hospitalization, and using the teach‐back method can address health literacy and decrease re‐presentation.^37^

### Strengths

Our study highlights the need for a holistic approach to understanding factors associated with ED visits and readmissions after head and neck surgery. A re‐presentation rate of 34.19% is high and highlights room for improvement terms of quality of care. Re‐presentation can result in financial toxicity for many Americans, not to mention the time and financial loss from missing work. For institutions, readmission results in penalties that can reduce Medicare payment to healthcare systems by up to 3% if readmissions exceed a given threshold.^38^ Our results provide avenues to help mitigate these individual and institutional‐level challenges by identifying key risk factors that can be addressed to reduce readmission rates and improve institutional and patient outcomes. We identified both clinical (tumor site), institutional (short PLOS), and social determinants of health (high ADI) factors. Due to the skewing of ADI toward higher levels in the head and neck cancer population, they may be especially sensitive to interventions that address ADI factors. It may be cost‐effective for hospitals and policymakers to invest in community outreach programs in high‐deprivation neighborhoods, particularly focusing on increasing resources and healthcare access.

Postoperative care and discharge planning followed standardized institutional protocols; however, the final discharge decisions were made at the discretion of the treating multidisciplinary care team in collaboration with the patient. Standard discharge planning included assessment of medical stability, wound and airway status, nutritional needs, pain control, and anticipated postoperative support requirements. Based on these factors, the care team determined the most appropriate discharge destination and level of post‐discharge services. While the overall framework for discharge planning was standardized, variability in patient complexity and social circumstances may have necessitated individualized decision‐making. Lastly, we chose a 60‐day outcome window to better reflect the complex postoperative time course after head and neck cancer surgery, as many complications and care needs emerge weeks after discharge and may not be captured within the traditional 30‐day timeframe.

### Limitations

Despite being one of the first studies to link ADI with ED visits and readmissions after oncologic HNS, our study has some limitations. While ADI is a well‐validated surrogate measure of disparities, it does not capture individual circumstances that contribute to these disparities. These include personal income, educational attainment, employment status, insurance stability, housing insecurity, transportation access, caregiver availability, health literacy, language barriers, or adherence to postoperative care instructions.^39^, ^40^ In addition, several important clinical and health system–level variables not fully captured include frailty, nutritional status, substance use, mental health comorbidities, outpatient follow‐up access, care coordination quality, discharge planning, home health utilization, and variation in surgeon or institutional practice patterns.^41^, ^42^ As a result, residual confounding related to these unmeasured or incompletely measured factors may remain despite adjustment in multivariable analyses.

The retrospective nature of the present study limits data to only those available in the electronic medical records. As a high‐volume tertiary academic center that treat patients from around the United States, our study does not account for patients who underwent surgery at our institution and later presented to the ED or were readmitted at an outside institution. It should be noted that while the odds ratio for length of stay was statistically significant and the 95% Cl did not cross 1, the effect size represents only a 2% reduction in odds per unit of readmission/ED‐visit. This is likely due to our large sample size which allows minimal differences to achieve statistical significance. Thus, statistical significance in this context may not translate to clinical significance and the true impact on patient outcomes may be limited. Our data on wound complications did not include wound classification and therefore, we could not do a more nuanced subgroup analysis investigating whether certain wound classifications were more likely to re‐present to the hospital. Although several predictors were statistically significantly associated with hospital re‐presentation, the magnitude of effect varied substantially. In the univariate models, discharge to post‐acute care facilities and advanced tumor stage demonstrated clinically meaningful effect sizes, whereas length‐of‐stay–related predictors showed small per‐unit increases in odds (1%‐3%). Despite statistical significance, this is unlikely to be clinically meaningful at the individual patient level and likely reflect underlying patient complexity and large sample size effects. Accordingly, LOS should be interpreted as a marker of clinical and social complexity rather than a modifiable driver of re‐presentation.

Furthermore, our predominantly White cohort represents the demographics of western Pennsylvania, limiting the generalizability of our findings beyond similar regions. However, this demographic serves as an exemplary setting to study disparities.

## Conclusion

Our study identified five key factors that increase patients' risk of representing to the hospital after oncologic head and neck surgery. Head and neck surgeons should be aware that patients with stage 4 tumors, laryngeal cancers, shorter PLOS, discharge to rehabilitation, nursing homes or with home health, and those residing in high ADI neighborhoods are at high risk of ED visits and readmission after primary discharge. To minimize the burden on the patient and the healthcare system, efforts to decrease PLOS must be balanced by unintended consequences of re‐presentation to the ED and readmission. Recognizing the importance of ADI provides additional opportunities to improved outcomes in head and neck cancer.

## Author Contributions

**Nana‐Hawwa Abdul‐Rahman**, conceptualization and design, data curation, statistical analysis, investigation, methodology, validation, writing and editing of manuscript; **Shaum Sridharan**, writing and editing of manuscript; **Matthew Spector**, conceptualization and design, data curation, methodology, supervision, validation, writing and editing of manuscript; **Carl H. Snyderman**, conceptualization and design, methodology, supervision, validation, writing and editing of manuscript.

## Disclosures

### Competing interests

None.

### Funding source

None.

## Supporting information

Supplementary Figure 1: Reasons for readmission/Emergency Department Visit.

Supplementary Figure 2: Histogram of the Frequency and Distribution of State Area Deprivation Index.

Supplementary Table 1: Time interval from discharge to representation and reasons for readmission.
Supplementary Table 2: Racial distribution and area of deprivation index (ADI)
Supplementary Table 3: Exploratory multivariable logistic regression: representation (ED‐visits and readmissions).

## References

[1] Kirby JB , Kaneda T . Neighborhood socioeconomic disadvantage and access to health care. J Health Soc Behav. 2005;46(1):15‐31. 15869118 10.1177/002214650504600103

[2] Sheetrit E , Brief M , Elisha O . Predicting unplanned readmissions in the intensive care unit: a multimodality evaluation. Sci Rep. 2023;13(1):15426. 10.1038/s41598-023-42372-y 37723231 PMC10507073

[3] Jencks SF , Williams MV , Coleman EA . Rehospitalizations among patients in the medicare fee‐for‐service program. N Engl J Med. 2009;360(14):1418‐1428. 10.1056/NEJMsa0803563 19339721

[4] Goel AN , Raghavan G , St John MA , Long JL . Risk factors, causes, and costs of hospital readmission after head and neck cancer surgery reconstruction. JAMA Facial Plast Surg. 2019;21(2):137‐145. 10.1001/jamafacial.2018.1197 30418467 PMC6439803

[5] Chen MM , Orosco RK , Harris JP , et al. Predictors of readmissions after head and neck cancer surgery: a national perspective. Oral Oncol. 2017;71:106‐112. 10.1016/j.oraloncology.2017.06.010 28688676

[6] Bur AM , Brant JA , Mulvey CL , et al. Association of clinical risk factors and postoperative complications with unplanned hospital readmission after head and neck cancer surgery. JAMA Otolaryngol Head Neck Surg. 2016;142(12):1184‐1190. 10.1001/jamaoto.2016.2807 27737442

[7] Stabellini N , Nazha A , Agrawal N , et al. Thirty‐day unplanned hospital readmissions in patients with cancer and the impact of social determinants of health: a machine learning approach. JCO Clin Cancer Inform. 2023;7:e2200143. 10.1200/CCI.22.00143 37463363 PMC10569782

[8] Kind AJH , Jencks S , Brock J , et al. Neighborhood socioeconomic disadvantage and 30‐day rehospitalization: a retrospective cohort study. Ann Intern Med. 2014;161(11):765‐774. 10.7326/M13-2946 25437404 PMC4251560

[9] Singh GK . Area deprivation and widening inequalities in US mortality, 1969‐1998. Am J Public Health. 2003;93(7):1137‐1143. 10.2105/ajph.93.7.1137 12835199 PMC1447923

[10] Kind AJH , Buckingham WR . Making neighborhood‐disadvantage metrics accessible—the neighborhood atlas. N Engl J Med. 2018;378(26):2456‐2458. 10.1056/NEJMp1802313 29949490 PMC6051533

[11] Gordon AM , Nian PP , Baidya J , Mont MA . A higher area deprivation index is associated with increased medical complications and emergency department utilizations after total hip arthroplasty. J Arthroplasty. 2025;40(24):1154‐1160. 10.1016/j.arth.2024.10.106 39490718

[12] Johnson AE , Zhu J , Garrard W , et al. Area deprivation index and cardiac readmissions: evaluating risk‐prediction in an electronic health record. J Am Heart Assoc. 2021;10(13):e020466. 10.1161/JAHA.120.020466 34212757 PMC8403312

[13] Shaw JH , Wesemann LD , Ayoola AS , Les CM , Charters MA , North WT . Comparison of area deprivation index, socioeconomic parameters, and preoperative demographics with postoperative emergency department visits after total knee arthroplasty. J Arthroplasty. 2021;36(8):2788‐2794. 10.1016/j.arth.2021.03.058 33902984

[14] Goldberg ZN , Jain A , Wu R , Cognetti DM , Goldman RA . Social determinants of health impact complications following free‐flap reconstruction for head and neck. Otolaryngol Head Neck Surg. 2025;172(1):91‐99. 10.1002/ohn.953 39189141 PMC11697525

[15] Graboyes EM , Cagle JL , Ramadan S , et al. Neighborhood‐level disadvantage and delayed adjuvant therapy in head and neck cancer. JAMA Otolaryngol Head Neck Surg. 2024;150(6):472. 10.1001/jamaoto.2024.0424 38662392 PMC11046410

[16] Balogun Z , Gardiner LA , Li J , Moroni EA , Rosenzweig M , Nilsen ML . Neighborhood deprivation and symptoms, psychological distress, and quality of life among head and neck cancer survivors. JAMA Otolaryngol Head Neck Surg. 2024;150(4):295‐302. 10.1001/jamaoto.2023.4672 38386337 PMC10884950

[17] Hu J , Bartels CM , Rovin RA , Lamb LE , Kind AJH , Nerenz DR . Race, ethnicity, neighborhood characteristics, and in‐hospital coronavirus disease‐2019 mortality. Med Care. 2021;59(10):888‐892. 10.1097/MLR.0000000000001624 34334737 PMC8446301

[18] Kitchen C , Hatef E , Chang HY , Weiner JP , Kharrazi H . Assessing the association between area deprivation index on COVID‐19 prevalence: a contrast between rural and urban U.S. jurisdictions. AIMS Public Health. 2021;8(3):519‐530. 10.3934/publichealth.2021042 34395702 PMC8334638

[19] Coblens OM , Brant JA , Thomas WW , Fischer JP , Newman JG , Cannady SB . American College of Surgeons National Surgical Quality Improvement Program assessment of risk factors for 30‐day unplanned readmission in patients undergoing head and neck surgery requiring free tissue reconstruction. Head Neck. 2020;42(2):230‐237. 10.1002/hed.25995 31674089

[20] Tucker J , Hollenbeak CS , Goyal N . Discharge destination and readmissions among patients with head and neck cancer. Laryngoscope Investig Otolaryngol. 2022;7(5):1407‐1429. 10.1002/lio2.890 PMC9575139 36262465

[21] Graboyes EM , Kallogjeri D , Zerega J , et al. Association of a perioperative education program with unplanned readmission following total laryngectomy. JAMA Otolaryngol Head Neck Surg. 2017;143(12):1200‐1206. 10.1001/jamaoto.2017.1460 28880984 PMC5824298

[22] Shenson JA , Craig JN , Rohde SL . Effect of preoperative counseling on hospital length of stay and readmissions after total laryngectomy. Otolaryngol Head Neck Surg. 2017;156(2):289‐298. 10.1177/0194599816671695 27677600

[23] Hu J , Kind AJH , Nerenz D . Area deprivation index predicts readmission risk at an urban teaching hospital. Am J Med Qual. 2018;33(5):493‐501. 10.1177/1062860617753063 29357679 PMC6027592

[24] Kellett W , Jalilvand A , Collins C , et al. Area deprivation index predicts mortality for critically ill surgical patients with sepsis. Surg Infect (Larchmt). 2023;24(10):879‐886. 10.1089/sur.2023.232 38079187 PMC10714256

[25] Basu S , Berkowitz SA , Phillips RL , Bitton A , Landon BE , Phillips RS . Association of primary care physician supply with population mortality in the United States, 2005‐2015. JAMA Intern Med. 2019;179(4):506‐514. 10.1001/jamainternmed.2018.7624 30776056 PMC6450307

[26] Virapongse A , Misky GJ . Self‐identified social determinants of health during transitions of care in the medically underserved: a narrative review. J Gen Intern Med. 2018;33(11):1959‐1967. 10.1007/s11606-018-4615-3 30128789 PMC6206338

[27] Kangovi S , Barg FK , Carter T , et al. Challenges faced by patients with low socioeconomic status during the post‐hospital transition. J Gen Intern Med. 2014;29(2):283‐289. 10.1007/s11606-013-2571-5 23918162 PMC3912302

[28] Ardekani A , Fereidooni R , Heydari ST , Ghahramani S , Shahabi S , Bagheri Lankarani K . The association of patient‐reported social determinants of health and hospitalization rate: a scoping review. Health Sci Rep. 2023;6(2):e1124. 10.1002/hsr2.1124 36846535 PMC9944244

[29] Larson NI , Story MT , Nelson MC . Neighborhood environments. Am J Prev Med. 2009;36(1):74‐81.e10. 10.1016/j.amepre.2008.09.025 18977112

[30] Coughlin SS , Vernon M , Hatzigeorgiou C , George V . Health literacy, social determinants of health, and disease prevention and control. J Environ Health Sci. 2020;6(1):3061. 33604453 PMC7889072

[31] Scarpato KR , Kappa SF , Goggins KM , et al. The impact of health literacy on surgical outcomes following radical cystectomy. J Health Commun. 2016;21(sup2):99‐104. 10.1080/10810730.2016.1193916 27661137 PMC5080660

[32] Trutner ZD , Furlough K , Martinez A , et al. Is health literacy associated with surgical outcomes? A systematic review. J Surg Res. 2023;291:720‐733. 10.1016/j.jss.2023.06.044 37572516

[33] Harley RJ , Atchison K , Li J , et al. Health literacy and adherence to clinical recommendations in head and neck cancer. Health Literacy Res Pract. 2023;7(1):e52‐e60. 10.3928/24748307-20230222-01 PMC9991085 36888985

[34] Sawaf T , Virgen CG , Renslo B , et al. Association of social‐ecological factors with delay in time to initiation of postoperative radiation therapy: a prospective cohort study. JAMA Otolaryngol Head Neck Surg. 2023;149(6):477‐484. 10.1001/jamaoto.2023.0308 37079327 PMC10119772

[35] Mitchell SE , Sadikova E , Jack BW , Paasche‐Orlow MK . Health literacy and 30‐day postdischarge hospital utilization. J Health Commun. 2012;17(Suppl 3):325‐338. 10.1080/10810730.2012.715233 23030580

[36] Ludwig J , Sanbonmatsu L , Gennetian L , et al. Neighborhoods, obesity, and diabetes—a randomized social experiment. N Engl J Med. 2011;365(16):1509‐1519. 10.1056/NEJMsa1103216 22010917 PMC3410541

[37] Pugh J , Penney LS , Noël PH , et al. Evidence‐based processes to prevent readmissions: more is better, a ten‐site observational study. BMC Health Serv Res. 2021;21:189. 10.1186/s12913-021-06193-x 33648491 PMC7919066

[38] Hospital Readmissions Reduction Program (HRRP). September 10, 2024. https://www.cms.gov/medicare/payment/prospective-payment-systems/acute-inpatient-pps/hospital-readmissions-reduction-program-hrrp

[39] Braveman P , Gottlieb L . The social determinants of health: it's time to consider the causes of the causes. Public Health Rep. 2014;129(Suppl 2):19‐31. 10.1177/00333549141291S206 PMC3863696 24385661

[40] McIsaac DI , Taljaard M , Bryson GL , et al. Frailty as a predictor of death or new disability after surgery: a prospective cohort study. Ann Surg. 2020;271(2):283‐289. 10.1097/SLA.0000000000002967 30048320

[41] Lee JH , Ba D , Liu G , Leslie D , Zacharia BE , Goyal N . Association of head and neck cancer with mental health disorders in a large insurance claims database. JAMA Otolaryngol Head Neck Surg. 2019;145(4):339‐344. 10.1001/jamaoto.2018.4512 30816930 PMC6481424

[42] Tyler N , Hodkinson A , Planner C , et al. Transitional care interventions from hospital to community to reduce health care use and improve patient outcomes: a systematic review and network meta‐analysis. JAMA Netw Open. 2023;6(11):e2344825. 10.1001/jamanetworkopen.2023.44825 38032642 PMC10690480

