# Supplementary material for: Clinical and Socioeconomic Predictors of 60‐Day Rehospitalization After Oncologic Head and Neck Surgery
Source: Otolaryngol Head Neck Surg. 2026 Feb 6;174(5):1270–8. doi: 10.1002/ohn.70164 (PMC13126435; doi:10.1002/ohn.70164)

Reason for Readmission/Emergency Department Visit
N= 372

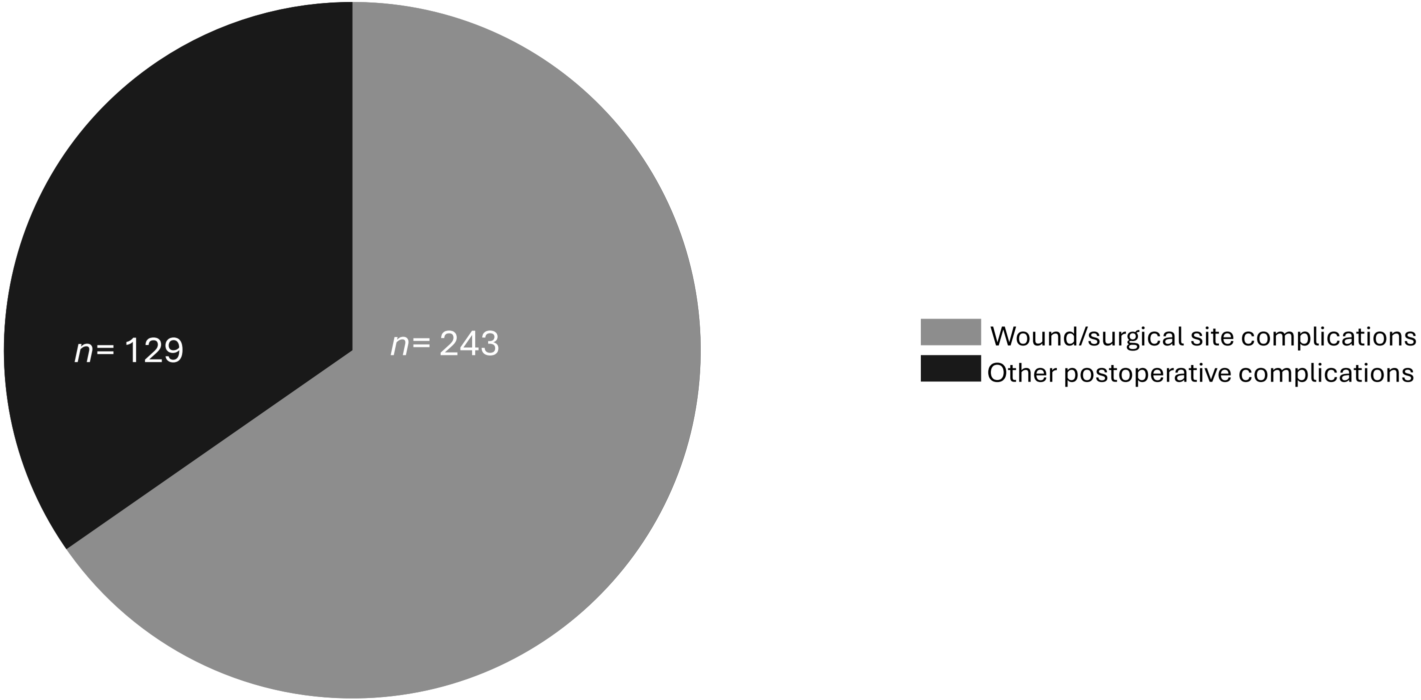

Supplement: Supplementary file 1 — Supplementary Figure 1: Reasons for readmission/Emergency Department Visit. [file OHN-174-1270-s002.pdf]
