# Supplementary figures and images for: Clinical and Socioeconomic Predictors of 60‐Day Rehospitalization After Oncologic Head and Neck Surgery
Source: Otolaryngol Head Neck Surg. 2026 Feb 6;174(5):1270–8. doi: 10.1002/ohn.70164 (PMC13126435; doi:10.1002/ohn.70164)

**Histogram of the Frequency and Distribution of State Area Deprivation Index**

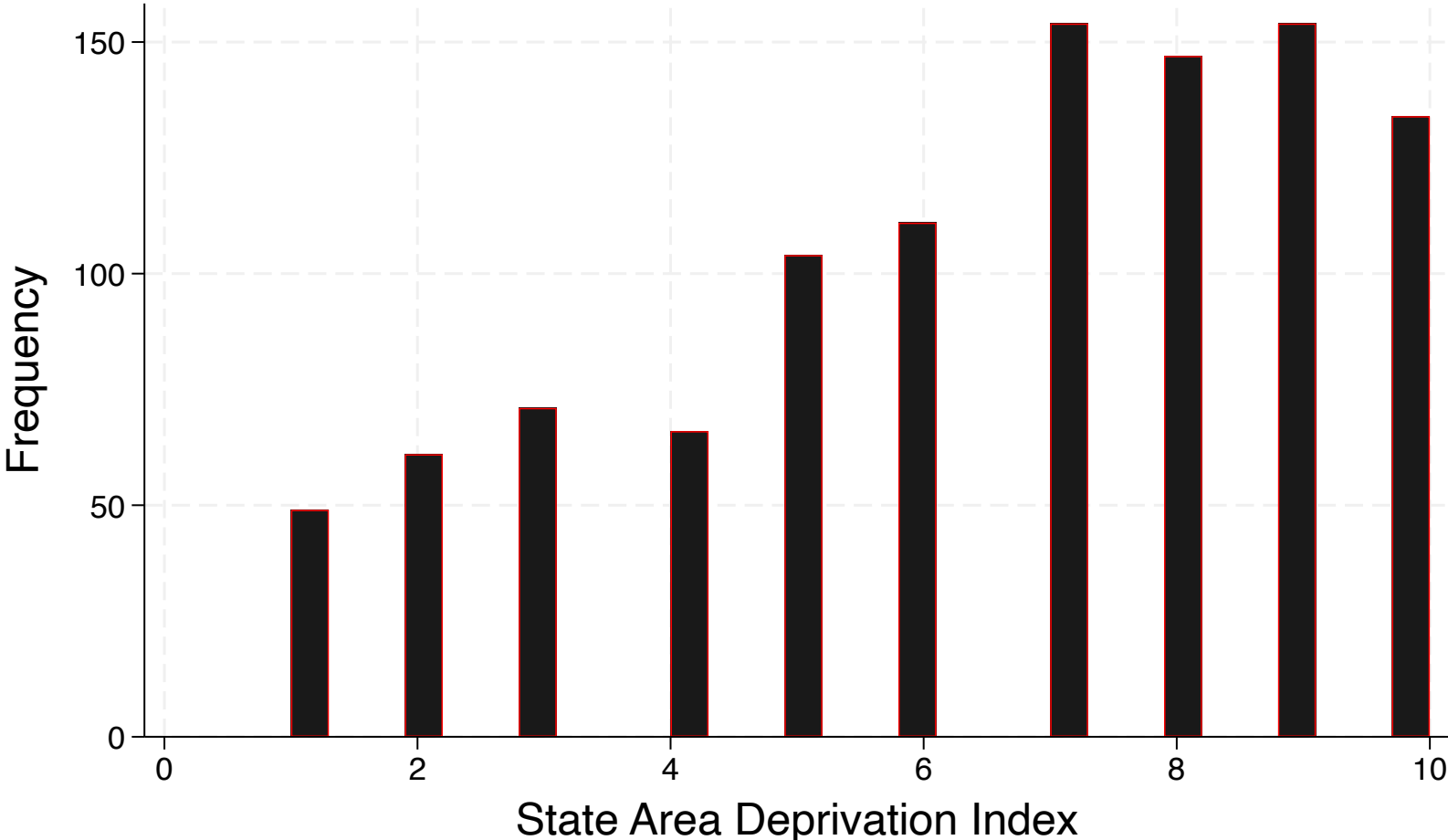

Supplement: Supplementary file 2 — Supplementary Figure 2: Histogram of the Frequency and Distribution of State Area Deprivation Index. [file OHN-174-1270-s001.pdf]
