# Supplementary material for: Clinical and Socioeconomic Predictors of 60‐Day Rehospitalization After Oncologic Head and Neck Surgery
Source: Otolaryngol Head Neck Surg. 2026 Feb 6;174(5):1270–8. doi: 10.1002/ohn.70164 (PMC13126435; doi:10.1002/ohn.70164)
Supplement: Supplementary file 3 — Supplementary Table 1: Time interval from discharge to representation and reasons for readmission. Supplementary Table 2: Racial distribution and area of deprivation index (ADI) Supplementary Table 3: Exploratory multivariable logistic regression: representation (ED‐visits and readmissions). [file OHN-174-1270-s003.docx]

**SUPPLEMENTARY TABLE 1: Time interval from discharge to representation and reasons for readmission**

| **Variable** | **Non-surgical site complications**  ***n*=** 243 | **Surgical site complications**  ***n*=** 129 | **p-value** |
| --- | --- | --- | --- |
| Time interval from discharge to readmission: days, *median (range)* | **20 (7 - 60)** | **10 (1 - 56)** |  |
| Represented within 7-days after discharge, yes: *n (%)* | **2 (5.13)** | **37 (94.87)** | **<0.001*** |
| No, *n (%)* | **241 (72.37)** | **92 (27.63)** |  |

IQR: Interquartile range

* Indicates significance: p<0.05

**Supplementary Table 2: Racial Distribution and Area of deprivation index (ADI)**

| **Variable** | **ADI (Quartile 1-3)** | **ADI (Quartile 4)** | **p-value** |
| --- | --- | --- | --- |
| Race: White: *n (%)* | 591 (60.37) | 388 (39.63) | <0.001* |
| African American and Asian | 14 (29.17) | 34 (70.83) |  |

* Indicates significance: p<0.05

**Supplementary Table 3: Exploratory Multivariable Logistic Regression: Representation (ED-visits and readmissions)**

|  | Readmission/Representation to ED | | |
| --- | --- | --- | --- |
|  | *Odds ratio* | *95% Cl* | *p-value* |
| Age at diagnosis | 1.00 | 0.99 - 1.01 | 0.781 |
| Sex |  |  |  |
| Male, Ref | - | - | - |
| Female | 1.11 | 0.82 - 1.49 | 0.509 |
| **Race** |  |  |  |
| White, Ref |  |  |  |
| Black or Asian | 1.03 | 0.54 - 1.94 | 0.930 |
| **Site** |  |  |  |
| Non-Larynx, Reference |  |  |  |
| Larynx | 1.56 | 1.09 - 2.18 | **0.014*** |
| **Tumor Stage** |  |  |  |
| 1, 2, 3, Reference | - | - | - |
| 4 | 1.29 | 0.98 - 1.71 | 0.072 |
| **Discharge Location** |  |  |  |
| Home, Reference |  |  |  |
| Home Health or other facilities | 1.97 | 1.41 - 2.76 | **<0.001*** |
| **State ADI, quartile** |  |  |  |
| 1, 2, 3, Reference | - | - | - |
| 4 | 1.35 | 0.91 - 2.02 | 0.140 |

ADI: Area of deprivation index

* Indicates significance: p<0.05
